# Sociobiology on Screen. The Controversy Through the Lens of *Sociobiology: Doing What Comes Naturally*

**DOI:** 10.1007/s10739-023-09719-7

**Published:** 2023-06-29

**Authors:** Cora Stuhrmann

**Affiliations:** LMU Munich, Munich, Germany

**Keywords:** Sociobiology, E.O. Wilson, Robert Trivers, Science for the people, Richard Lewontin, Stephen Jay Gould

## Abstract

When the sociobiology debate erupted in 1975, there were almost too many contributions to the heated exchanges between sociobiologists and their critics to count. In the fall of 1976, a Canadian educational film entitled *Sociobiology: Doing What Comes Naturally* sparked further controversy due to its graphic visuals and outrageous narration. While critics claimed the film was a promotional tool to further the sociobiological agenda in educational settings, sociobiologists quickly distanced themselves from the film and, in turn, accused the critics of consciously misrepresenting sociobiology by organizing showings of the film. Using audio, video, archival, and published sources, this paper explores the complicated history of *Sociobiology: Doing What Comes Naturally* and demonstrates how the public debate about the film reflects the positions, polemics, and polarization of the sociobiology debate as a whole.

“Which one was that young guy with the mustache?” an audience member wondered after the film had ended with the sound of machine guns and military boots and the murmurs and giggles had died down. “That was Trivers.”—“And the other one?” Freda Salzman answered with some snark in her voice: “The gray-haired one was DeVore, and the one with the ants was Wilson. In fact, that’s what Wilson has done most of his work on: ants!” She got her reaction when the reply was interrupted by laughter at the mention of Wilson’s use of ants as a model for the very human behaviors explored in the film, behaviors touching on cultural issues of the day, such as “sex roles” and “man’s” proclivity for war. A lengthy discussion about sexism in science, human nature, and biological determinism ensued that November evening in 1977 at an MIT seminar room.^1^

Freda Salzman was a physics professor, local activist, and member of Science for the People and the Women, Science and Social Control Collective. Her activism against sexism was informed by a painful legal battle with her employer, the University of Massachusetts, which she finally won when she received tenure in 1975.^2^ Two years later, when Salzman was invited to speak as part of the anarcho-intellectual “Black Rose” lecture series,^3^ she decided to speak about “Scientific Sexism: ‘From Freud to Sociobiology’” (Fig. 1) and to illustrate this issue by showing a film: *Sociobiology: Doing What Comes Naturally (*hereafter *Naturally)*.^4^ To her, and to many in the audience, it epitomized the reactionary and sexist outlook of the new discipline of sociobiology. The film was understood to promote sociobiology through interviews with the Harvard academics mentioned in her answer: Professors E.O. Wilson, a distinguished entomologist and professor of biology, and Irven DeVore, a popular professor of social anthropology and primatology, whose work focused on baboon social life. The third interviewee was, as the film introduced him, “super theorist Robert Trivers” (Judd 1978, p. 16; Kurland 1983, p. 267), a former graduate student in the biology department at Harvard, who had by the time of the film’s release moved up to assistant professor. The film featuring these three interviewees was to become one of the most vividly remembered artifacts of the sociobiology debate.

**Fig. 1:**
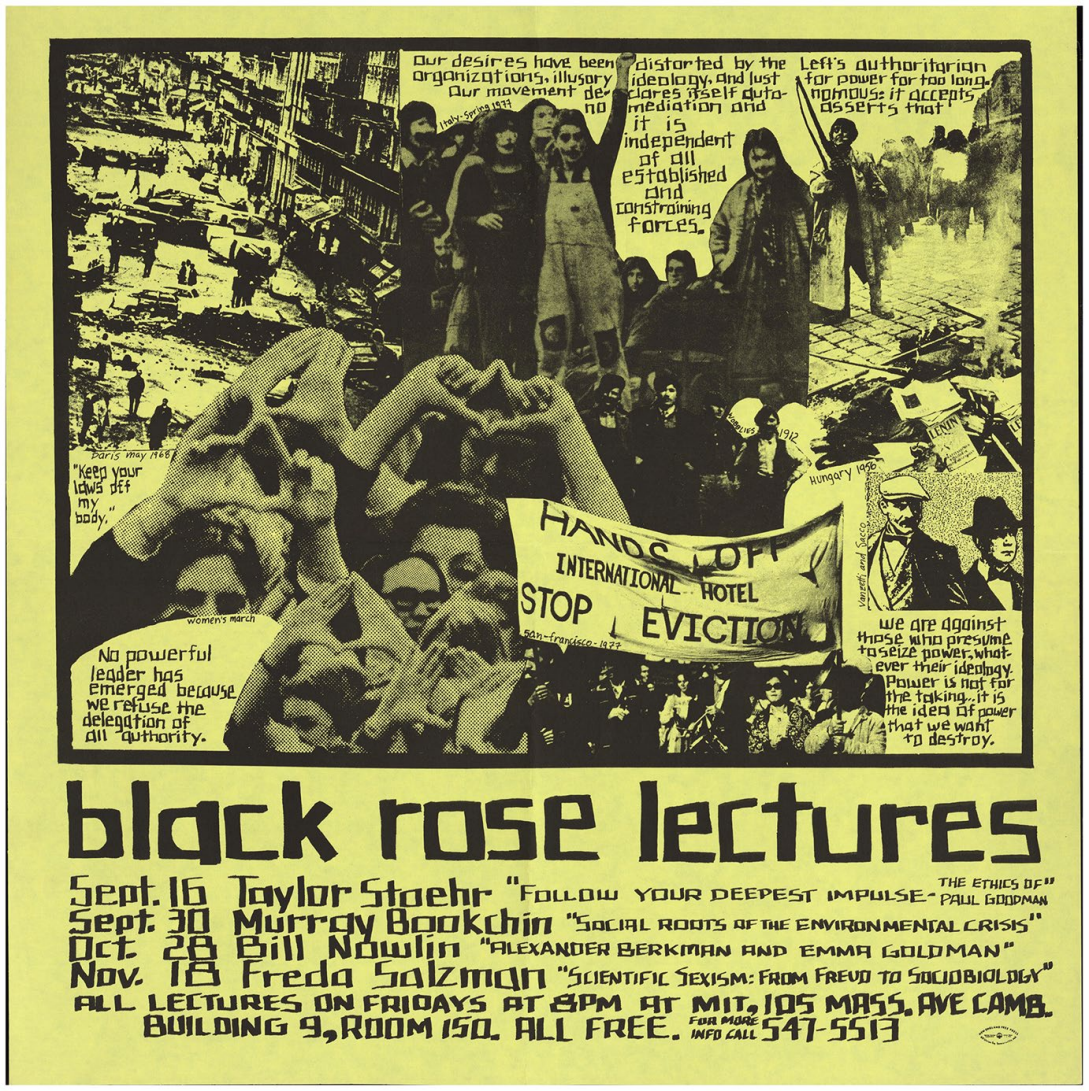
Poster for the Black Rose Lectures in the fall of 1977 with Freda Salzman’s talk on “Scientific Sexism. From Freud to Sociobiology.” (“Black Rose Lectures,” poster, fall 1977, SCLP0827, Joseph A. Labadie Collection, Special Collections Research, University of Michigan Library)

Many contemporaries still remember this film today, possibly because it was a notable exception in a debate that was dominated by the written word. After the publication of E.O. Wilson’s *Sociobiology: The New Synthesis* (Wilson 1975a), the critics had conveyed their concerns in letters to the editor and position papers and the parties had fought by publication through scathing reviews and bitter rebuttals. The flurry of academic publications was flanked by dozens of newspaper and magazine articles covering the scientific controversy, often including interviews and input provided by the parties. The continuous flow of publications and press coverage encouraged and sustained public interest in sociobiology and its critics for several years after 1975. A high level of public engagement that led to increased polemics and polarization had already become a main characteristic of the sociobiology debate. The film’s immediate impact was to accelerate this already existing dynamic. This was due to the film’s mixture of over-sensationalized narration and crass imagery, exemplary of a style of popular science filmmaking in the 1960s and 1970s, that straddled the line between education and entertainment. With more productions by private companies pursuing commercial success, science content on TV was pulled into the direction of social relevance and sensationalism (LaFollette 2013, pp. 101–120). To maximize the profitability of footage, TV production companies planned multivalent films, intended to play in front of multiple audiences from children, students and interested laypeople to scientific experts.^5^ In the case of *Naturally* the intended audiences were Canadian TV viewers and high school and college students, but it became an unexpected audience favorite in the circles of radical scientific activism.

Freda Salzman was not the only academic activist using the film to expose larger issues with sociobiology as a discipline. Different radical groups, the most prominent being Science for the People, showed the film widely, especially in the Boston area, in the years after its release in 1976. The fact that the showings were organized by leftist critics of sociobiology begs the question: If *Naturally* was supposedly a pro-sociobiology film, why were sociobiology’s most fervent critics the ones to show and popularize it? As the film was presented as an extension of sociobiological reasoning, a dangerous tool of sociobiological advocacy, and a representation of sociobiologists’ views, the common assumption became that the film was somehow authored or at least authorized by leading sociobiologists. This demonstrates how the film quickly became subsumed into the polarized atmosphere of the sociobiology debate: Since sociobiology’s critics were against the film, the sociobiologists must be behind the film somehow.

Sociobiologists were, however, desperate to distance themselves from this film with its flashy visuals and overbearing narration, as its sensationalistic tone alone was enough to undermine the scientific legitimacy of sociobiology. In fact, the reaction to the film was one of the few points of agreement between the sociobiologists and their critics. Both sides of the debate hated the film. One would assume, then, that the film would be removed from circulation, but instead frequent showings became a staple of anti-sociobiological activism. The reason: the film was so bad, it was good for critics. Merely showing the film did a lot of the heavy argumentative lifting for the group, because it showed sociobiology’s sexist and backward attitude about the division of labor between men and women and its violent and aggressive understanding of human nature. They considered *Naturally* as the gift that kept on giving, and it became a centerpiece of their activism and resistance to sociobiology.

Both at the time and still today, there remained a certain mystery about who had produced the film and to what end. John Tooby, a foundational figure in evolutionary psychology, was also a graduate student of DeVore’s at Harvard during the debate and recalled being present at the first screening of the film by Science for the People in mid-December 1976. When I brought up the film during an interview I conducted with him, the first thing Tooby said was: “I can’t tell you something crucial, which is who made it.”^6^ Similarly, about half an hour into the discussion session after the film in 1977, a female audience member asked Freda Salzman about its origins. “Well, that we don’t know, we do know as recently as a few weeks ago … ”—five seconds into Salzman’s answer, the recording cuts off, and we are left in the dark about what Salzman knew regarding the film’s origins. However, the true origins of the film were not as sinister as either side assumed. It was neither created as a pro-sociobiology propaganda film nor was it intended as a leftist attack piece; it was simply an unfortunately produced made-for-TV documentary.

In order to explore how this film influenced and exemplified the larger dynamics of the sociobiology debate, it is necessary to understand the strong reactions it evoked by performing a close reading of the film.^7^ However, this is complicated by the fact that the original film reels are lost.^8^ I reconstruct the lost film by comparing a later re-edited version with reviews of the first version,^9^ as well as the audio recording of Freda Salzman’s lecture, which starts with the last five minutes of the film. This will give an idea how the film’s particular mixture of interviews with leading scientists with strong narration, imagery, and sound effects left a powerful impact on its audience. I will use the reconstructed film as a central anchoring point from which I expand into a range of materials pertaining to it, from archival sources to newspaper articles, from old audio recordings to recent oral history interviews. This reconstruction is necessary as I argue that the history of the film provides an entryway into understanding the sociobiology controversy as it is a microcosm of the positions, polemics, and polarization that developed in the public debate about sociobiology as a whole.

## A Public Debate: Synthesizing the Political, the Personal and the Disciplinary Dimensions of Sociobiology

The relative influence of political, personal, and scientific agendas behind both sociobiology and its critique has been a contested topic in both the sociobiology debate itself and its scholarly analysis. The events of the sociobiology debate within academia are most thoroughly documented in Ullica Segerstråle’s comprehensive *Defenders of the Truth* (Segerstråle 2000),^10^ where she delves into the motivations of the main Harvard antagonists, Richard Lewontin and E.O. Wilson. The conflict between them was not primarily due to politics, she asserts, but differences in their personal “scientific-cum-moral agendas” (Segerstråle 2000, p. 35). While Wilson favored pursuing inspiring, but speculative hypotheses, Lewontin argued true scientific rigor required sticking to well-established and evidence-based claims. She is part of a group of scholars (some with close associations to Wilson) that emphasize a non-political interpretation of the debate. Segerstråle stresses that deeply held scientific convictions caused irreconcilable differences between Wilson and his critics. Others in this group, such as Michael Ruse, look instead to differences in personality and religious background to explain the intensity of their disagreement (Ruse and Gibson 2015, pp. 257–258). In his autobiography *Naturalist,* Wilson himself promoted a similar understanding of the debate and adopted Segerstråle’s interpretation (Wilson 1994, chap. 17).^11^ In the chapter devoted to the sociobiology debate, Wilson painted a picture of a clash of personalities between himself and Richard Lewontin. A recent biography by the popular science author Richard Rhodes is based on extensive interviews with Wilson and follows Wilson’s own account closely. Additionally, he suggests Lewontin’s professional jealousy and ego as other driving forces (Rhodes 2021, chap. 9). Lewontin objected to such interpretations in a letter to Wilson as soon as they were first suggested by Wilson in 1975: “While it is the style at Harvard to try to place all disagreements on matters of substance in terms of a struggle between personalities (what my wife calls the ‘High Noon at Harvard syndrome’) that is most certainly not the case.”^12^ While Lewontin stressed substantive issues, Wilson may have been motivated by personal ambitions. Charlotte Sleigh details how Wilson pivoted his research focus to theoretical biology after an unsuccessful foray into the depths of ant taxonomy. In doing so, she argues, Wilson used ants to “establish himself as a master of one area, ready to move out to his big synthetic theory” (Sleigh 2007, p. 216).

Leaving personalities, beliefs, and ambitions aside, other scholars regard the sociobiology debate as an expression of political tensions and a fundamental socio-political reorientation that occurred in the 1970s (Jumonville 2002; Yudell and DeSalle 2000). Despite the shock of John F. Kennedy’s assassination in 1963, optimistic political and social goals were pursued in its aftermath, such as the introduction of affirmative action and the war on poverty as part of the Great Society programs, initiated by Lyndon B. Johnson. But the idea that such goals were achievable through political measures had eroded by the early 1970s (Schulman 2002; Berkowitz 2005). Stagflation and the oil crisis led to an economic crisis, increased knowledge of atrocities committed by US troops and wide-spread opposition to the Vietnam war caused a moral crisis, and finally, a political crisis emerged as trust in government fell dramatically after the revelation of the Watergate scandal and Richard Nixon’s resignation. Therefore, the mid-1970s have been characterized as the beginning of an “Age of Fracture” (Rodgers 2012, p. 3), realigning the political landscape in the US. As a result, the progressive camp internally divided, marking the end of the liberal consensus of the 1960s; the New Left, as well as a conservative, sun-belt-supported majority under Nixon, emerged as dominant political groups (Kruse and Zelizer 2019; Borstelmann 2012; Rodgers 2012; Perlstein 2010; Berkowitz 2005; Schulman 2002). This development corresponds with a politico-generational divide that is often cited as an important factor in the sociobiology debate: While Gould and Lewontin embodied the values and activist tactics of the New Left, Wilson is characterized as a member of earlier generations of liberals that stressed civility and consensus. (Sheldon 2014; Jumonville 2002).

In addition to these generational tensions, local political events such as a high level of campus activism, social unrest around the practice of bussing (school integration), and recent debates on racial differences in intelligence contributed to the especially heated atmosphere on Boston campuses when sociobiology first appeared (Moore 2008). Understanding the sociobiology debate as having mainly political roots aligns with early interpretations of the debate as political (Rensberger 1975b). Problematically, this comes close to echoing Wilson’s own assertion that the attacks on sociobiology were merely driven by New Left political activism and had no scientific basis. Wilson’s strategy was to contrast his own actions as driven by science with his opponents’ actions which were merely driven by political ideology. However, this minimizes both Wilson’s disciplinary ambitions and the scientific nature of the critique.

Sociobiology’s appearance also belongs to a historical narrative of disciplinary developments in biology. The publication of *Sociobiology* came at the end of a phase of popular appeal of evolutionary biology and ethology to explain human behavior, especially aggression, in the 1960s (Milam 2018; Weidman 2021). In this vein, Wilson repeatedly described his vision for sociobiology as a new and revolutionary discipline that would provide a biological basis for the social sciences: “It may not be too much to say that sociology and the other social sciences, as well as the humanities, are the last branches of biology waiting to be included in the Modern Synthesis” (Wilson 1975a, p. 4). In Wilson’s view, using the Modern Synthesis of the 1930s and 1940s as the theoretical basis made sociobiology a counterprogram to the looser evolutionary theorizing of the ethologists of the 1960s, such as Konrad Lorenz and Robert Ardrey. It expanded the reach of evolutionary biology and specifically the synthetic theory of evolution into the social sciences. Underlying this expansion was the belief that a synthetic theory of evolution, unifying explanatory levels from the gene to the human, is achievable (Smocovitis 1992, pp. 226–227).

It also functioned to increase the relevance of evolutionary biology vis-à-vis molecular biology.^13^ In his autobiography, Wilson recounted the perceived threat by molecular biology, represented by James Watson, to evolutionary biology and the ensuing inner-departmental struggle at Harvard under the title “The Molecular Wars” (Wilson 1994, Chapter 12; see also: Sleigh 2007, Chapter 9). Wilson saw the situation at Harvard as representative of larger dynamics that undermined the status of organismic and behavioral disciplines within biology and looked for disciplinary solution: combining recent advances in evolutionary theory with a focus on organisms and their behavior promised continued scientific and public relevance. It also allowed an extension into the social sciences if non-human and human behavior was compared and classified based on a common evolutionary understanding. Wilson’s critics opposed this disciplinary extension, based on what they deemed to be a hyper-adaptationist understanding of evolution (Gould and Lewontin 1979). Their efforts were part of an inner-biological movement that placed the Modern Synthesis under exceeding scrutiny and undertook efforts to reformulate it, ushering a period of debate and re-evaluation lasting from 1974 to 1987 (Smocovitis 1996, pp. 33–44).

The historiography of the sociobiology debate balances these different underlying dimensions—the personal, the political and the disciplinary. Shifting away from analyzing the reasons for the debate to analyzing its impact reveals the implicit consensus of these dimensions: Sociobiology was a public debate. Shortly after the initial controversy in the early 1980s, scholars focusing on rhetoric of science analyzed the strategies and narratives employed by Wilson and his critics in order to maximize public appeal of their positions (Lyne 1983; Journet 1984; Lyne and Howe 1990, 1992; Myers 1990; Ceccarelli 2001). At the end of his extensive analysis of the sociobiology debate, Greg Myers concluded that a resolution was impossible, and even unwanted: “Neither side seems particularly interested in persuading anyone who does not already agree with them. … In this sense the debate never seems to get anywhere, and yet it goes on and on.” (Myers 1990, p. 243). What Myers described here are the dynamics of public polarization, such as repeatedly stating and re-stating the same positions, appealing to only a certain subset of the public, eventually leading to a split in the public perception of the topic at hand. These dynamics of public polarization of the larger debate are also evident in the history of *Naturally*.

## The Background: From Scientific Controversy to Public Debate

The sociobiology debate that provided the backdrop to Salzman’s film discussion started shortly after Wilson published *Sociobiology: The New Synthesis* in June 1975 (Wilson 1975a). By the early summer of 1975, Wilson was in full swing in his outreach to popular audiences as *Sociobiology* was about to be published, presenting it as revolutionary and relevant with explanatory power for animal and human social behavior. Wilson’s almost 700-page tome was intended as a grand synthesis of recent advances in animal behavior studies on social behavior. Wilson felt his material on animals and his grasp of the underlying theories and models was strong enough to include a last chapter on humans entitled “Man. From Sociobiology to Sociology,” analyzing human behavior through an evolutionary lens and advocating for a biologizing of disciplines such as anthropology, sociology, or psychology.

Wilson’s ambitions with sociobiology were eagerly promoted in the press. In May 1975, the article “Updating Darwin on Behavior” appeared on the cover of the *New York Times* (Rensberger 1975a). An impressive feat, as even ground-breaking scientific discoveries rarely made the cover. The article itself touted sociobiology as a “new field of scientific inquiry” with “the revolutionary implication that much of man’s behavior toward his fellows, ranging from aggressive impulses to humanitarian inspirations, may be as much a product of evolution as is the structure of the hand or the size of the brain” and Wilson’s book as the “long-awaited definitive book on the subject,” its advance copies having “stimulated considerable excitement.” The *Boston Glob*e goes even further in accentuating sociobiology’s importance: “Wilson hopes for a wide audience for the book because he believes mankind is at a crucial point in its social evolution … . He thinks that sociobiology can offer some guidance and some warning signs in this transition phase” (McCain 1975, p. A4). Wilson himself authored the long article “Human Decency is Animal,” presenting the core tenets of sociobiology as using “comparison(s) of societies of different kinds of animals and of man … to devise and to test theories about the underlying hereditary basis of social behavior” with the intended goal that “sociobiological explanations … will, at the very least, provide perspective and a new sense of philosophical ease about human nature” (Wilson 1975b, p. 38). Wilson’s vision captured the public imagination by inviting the reader to witness the emergence of a new scientific understanding of human nature.^14^

Wilson’s success as a public scientist relied on his lively scientific prose. Ironically, his ability to charm a general reader also ignited the fiercest opposition he was to encounter in his career. As Jonathan Beckwith recalls in his autobiography (Beckwith 2002, pp. 137–138), the positive early coverage spurred a Boston group of radical biologists, including Richard “Dick” Lewontin and Stephen Jay Gould, into action, with Lewontin being especially skeptical of Wilson’s disciplinary ambitions: “He told me that Wilson had talked for some time of launching a major new effort to establish genetics and evolutionary theory as a basis for explaining human societies. Dick was skeptical of these claims, as he had been of earlier genetic theories of human behavior. We agreed to call together a few people who might share our concerns. We would meet to consider whether and how to respond to this public surfacing of sociobiology” (Beckwith 2002, p. 139). His local opponents consolidated into a study group of the book and affiliated themselves with Science for the People.^15^

This meeting was the foundation of the Sociobiology Study Group of Science for the People (hereafter SftP).^16^ One of their core convictions was that science, especially biology and medicine, plays an active role in shaping society’s expectations and therefore has a responsibility to advocate anti-racist and anti-sexist messages. They disagreed with the traditional understanding of science as an apolitical force in society, arguing that such an understanding was naïve and truly apolitical science impossible. They were concerned about the legitimacy of science being used as a resource to support reactionary policies. SftP viewed Wilson’s new discipline as an attempt to codify stereotypes by basing them on biology. They shared the aim of revealing sociobiology’s political and social implications and depriving it of scientific legitimacy. Their first meeting states their mission “to write a critique of the field of sociobiology in order to make the subject controversial.”^17^

Controversy was exactly what they did create. By the fall of 1975 they had leveraged the popular appeal and academic reach of their most prominent members, Stephen Jay Gould and Richard Lewontin, Harvard professors of paleontology and population genetics respectively, into a letter responding to a positive, if not entirely laudatory, book review by developmental biologist C.H. Waddington in the *New York Review of Books*. (Waddington 1975). Their letter titled “Against Sociobiology” (Allen et al. 1975) placed sociobiology in the long history of biological determinism,^18^ one that included Herbert Spencer’s Social Darwinism, the eugenics movement, the gas chambers of “Nazi Germany,” and Konrad Lorenz’ more recent ethology. They forcefully expressed their concern about sociobiology’s political implications: since behaviors such as primary child-care by women or territorial violence by men was naturalized, this provided a biological legitimacy to the societal status quo. Wilson was surprised, outraged, and hurt by the letter, written without warning behind his back by colleagues who even worked in the same building, and responded in kind, accusing the critics of political posturing and academic vigilantism (Wilson 1975c). The authors of the letter wanted to convince readers that sociobiology was not a revolutionary, rigorous, or legitimate science of human nature, but reactionary and pseudoscientific speculation intended to appeal to base prejudice, justify an unjust society, and invite dangerous political consequences. In contrast, Wilson argued that sociobiology was a proper science that rightly claimed legitimacy and that the political implications were put there by the opponents themselves to discredit the field by means of political activism rather than scientific research. With that, the battle lines were drawn.

Following this exchange, the controversy spread over the pages of major newspapers and science journals as well as across American campuses, with the group lecturing about the dangers of sociobiology while Wilson defended his discipline as well as his reputation. In the volatile political climate of the mid-1970s, academic polarization around sociobiology was achieved with astounding speed.

## Proudly Presenting Sociobiology: The Production of an Educational Film

In March 1972, a film team from Hobel-Leiterman Productions came to the Harvard campus to interview the leaders in a new field of science.^19^ The company specialized in documentaries for TV and classrooms and had already produced the acclaimed series “The Fabulous Sixties” (1969–1970, CTV). Their follow-up was to be “Here Come the Seventies” (1970–1973, CTV). Like its predecessor, it focused on social issues of the day, such as “Sex: Breaking Down the Barriers,” “Race Relations: Getting It Together,” or “Women: The Hand that Cradles the Rock.” It also included installments on science, medicine, and technology. The interview material collected at Harvard was intended for one such installment in this series “that looked ahead at anticipated technological marvels and innovations and did so with great flair and élan. The off-beat mood for each episode was set right from the opening credits, which featured a naked woman walking into a lake” (Wedge 2002).^20^

At the time of the interviews, E.O. Wilson rarely passed up a chance to promote his ideas. He had already presented his vision of a new discipline in an article for the *American Scientist* entitled “The Prospect for a Unified Sociobiology” (Wilson 1971a)^21^, and a documentary could help address a popular audience. While Wilson was the principal figure in the sociobiology debate, this was not the case in the film; prominently featured as fellow “sociobiologists”^22^ were DeVore, who had come to appreciate the evolutionary analysis of primate social behavior, and Trivers, whose insights along with those of William Hamilton and George Williams formed the theoretical basis of the kin-selectionist revolution within evolutionary biology.^23^

I would like to provide a detailed summary of the film itself. However, there are two versions of it that were in circulation in the late 1970s. The version employed by SftP was the original, 24-min cut, copyrighted in 1974 by Hobel-Leiterman Productions, on celluloid. The version I base my description on and the only one currently available is a recut 1978 version that is only 21 min long and distributed on VHS.^24^ However, several reviews allow me to summarize the original film while pointing out the most notable differences in tone and content to the recut version.^25^ The film is structured around interviews with Trivers, DeVore, and Wilson, each featured for about four to five minutes total. The interview footage is interspersed with stock footage set to narration in the style of what one reviewer calls the “Marlboro Man” (Kurland 1983, p. 267), with a deep-voiced narrator. In general, the recut version reduces the role of the narrator and is “noticeably ‘toned down’—the loud, driving rock music had disappeared as had the shots of long-haired street people and the sexist ‘angles’ (e.g., a woman in hot pants, a woman disrobing, provocative camera angles, etc.)” (Fritz 1980, p. 99). I will recount the film’s topics, interview statements, and concrete examples of scenes removed in the recut version of the film.

In the introductory segment, the narrator describes sociobiology as a new biology that will help humanity shape its future, and Trivers explains the concept of applying natural selection to human behavior. The original version then shows “full-screen shots of the wriggling *glutei maximi* of two tightly blue-jeaned females walking down the same Toronto street” (Kurland 1983, p. 267). Following the introduction, the first section of the film is concerned with reproduction. DeVore stresses the birth control pill as revolutionary in changing sex roles. Removed is a scene where “several hippie couples move from an open discussion of the new sexual freedom to pulsing courtship. The male member of one couple declares that ‘some females seek a husband, and others seek orgasms’” (Kurland 1983, p. 267). Next up in the recut version are college students interacting. In the original, Trivers is introduced as believing “that the sexes are biologically programmed or wired to do almost everything they do.”^26^ In the recut version, Trivers merely explains that female “vulnerability” to pregnancy caused behavioral preferences for emotional attachment before sex. He feels strongly that there is a “biological component” and sees this as opposed to an “element of the women’s liberation that feels quite the other way around … that sexual behavior merely reflects the larger oppression, etc. etc.” The original shows footage “that implies that women do not enjoy sex”^27^ and “of disrobing fashion models and young women in hot pants. The focus is quite deliberately on the pelvic area” (Judd 1978, p. 16).

The next section deals with males’ position in society. DeVore: “You don’t have to be a scientist to notice that among humans, men are much more interested in status and in politics than women are.” Is this due to biological background or social institutions? DeVore points to biology as male competition for status occurs in all animals. “The narrator tells us that ‘The message for males isn’t subtle and it isn’t subliminal; get out there and fight for your life … go for yards, dig, be a home ground hero.’ Football players, boxers and fighting baboons are spliced next to pictures of sexy women and the words ‘Possess Her’ flash on the screen” (Judd 1978, p. 16). In the recut version, this is replaced by the phrase “Be Somebody.” DeVore explains male competition for status and the development of hierarchies to curtail fighting. In humans, they take subtler forms such as clothing or accents, as shown by a montage of clips with different English accents signifying social status.

The following section explores the genetic roots of behavior. The narrator asks, “How far back can we go in understanding the origins of our behavior? … The dream: to connect the behavior of lower life forms and project them to understanding behavior in man.” This statement introduces Wilson, who wants to classify human social behavior to find its evolutionary origins and meaning. To the question, “Is there hope to find general qualities of behavior? … The answer is a very limited and qualified yes.” Sociobiology, as a discipline, aims to produce laws to predict and define in mathematical and quantitative terms common behaviors from ants to monkeys and even man. Wilson explains the importance of cooperative hunting for the evolution of human social life, and DeVore accordingly interprets the youth revolt as a revolt for more cooperative living. Again, the narrator’s role is reduced in the recut version; in the original, he asks: “Is it possible that in the next century we will look back at the youth revolt of today as part of our natural evolution?”.^28^

The final section focuses on war and conflict. After Trivers explains parent-offspring-conflict, the narrator transitions to warfare: “Is it possible our own inner biological clocks have been sending men to battle since the beginning of time? In our own thermonuclear age, we can no longer afford the ritual of war.” Trivers explains how using superior technology and warfare is genetically advantageous for reproductive success, which results in “certain genetic predispositions towards warfare. Now that’s not to say that we’ve got a gene to run off and slaughter each other every week. And that’s not saying that you can’t arrange socialization and international arrangements such as to do away with warfare someday, but you’ve got to face the fact that there are predisposing factors.” This is set to a montage of military marches and Vietnam war footage, including shots of dead bodies in the undergrowth. In the original, Trivers’s statement is additionally overlaid with loud machine gun fire that can be heard on the recording of Salzman’s lecture. Trivers then explains that classical warfare entailed “to grab up the women and you either inseminate them on the spot, or you take them back as concubines. … So I think warfare has traditionally had a strong sexual counterpart to it, which is certainly biological, and you don’t have to look far to see that there’s that tendency running today.”

Trivers then summarizes the ambitions of sociobiology, quoted here in full, over scenes from a contemporary Western city, likely 1970s Toronto: “It’s time we started viewing ourselves as having biological, genetic, and natural components to our behavior. And that we should start setting up a physical and social world which matches those tendencies and not assume that our behavior is infinitely malleable and therefore we can set up the world so as to produce the grossest national product regardless of how people actually live.” The narrator asks in the original: “Is man just another endangered species? Perhaps. But we are the first animals that have the power to consciously control whether we survive or not.” In both versions, he concludes sociobiology may give us a clue to “man’s own basic biological wiring.” The recut version ends; the original adds ominously: “When we look at where we’ve been, nuclear man is only an afterthought,” while the “upbeat, really ‘with it’ rock soundtrack featuring xylophones” (Kurland 1983, p. 267) plays us out.

Program announcements in Canadian newspapers suggest that *Naturally* was broadcast in 1972 and 1973. It did not cause much of a fuss: “Program probes human behavior” stated the TV guide—hardly controversial fare. Wilson and the others later claimed that they did not hear anything from the production company regarding the broadcasting and assumed the project had been scrapped.^29^ When Wilson and the others gave the interviews in 1972, there was no sociobiology debate. The tone taken in the interviews was not shaped by the controversy yet and, therefore, is less considered and careful, more speculative and candid. The interviews conveyed the sense of excitement of being on the verge of a scientific breakthrough, and the hope and enthusiasm this caused are palpable (Wilson 1994, chapter 16). However, when the film came out as part of the educational program of Hobel-Leiterman Productions in the fall of 1976, sociobiology had already been publicly attacked and criticized for over a year. When a seemingly “new” film appeared out of the blue, displaying similar aspirations as the book in the summer of 1975, it went counter to the more defensive tone the sociobiologists had employed in early 1976 and seemed like a provocative doubling down on their position. Time had marched on mercilessly during the controversy; after only four years, the interviews had already aged very badly. It is not surprising, then, that these 1972 interviews backfired for the sociobiologists in unexpected ways in the fall of 1976. In the arsenal of sociobiology’s opponents, the interviews became a crucial weapon to expose the true nature of sociobiology as genetic determinism and naïve reductionism.

## Outraged by Its Sexism and Racism: From Analysis to Action

**Fig. 2:**
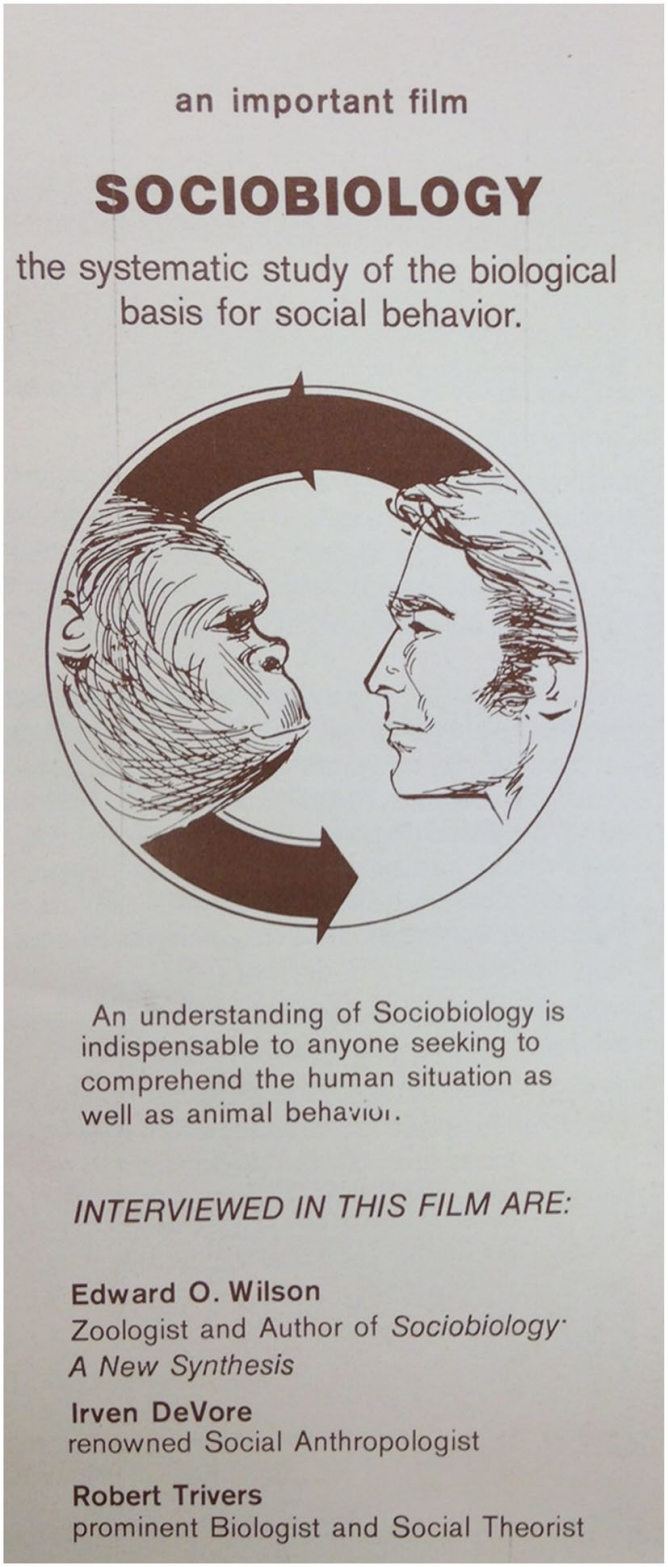
Front cover of leaflet advertising *Naturally* as an “important film.” These were distributed in the fall of 1976. (Document Associates, Leaflet for *Naturally*, Box 184, Edward O. Wilson Papers, Manuscript Division, Library of Congress. Courtesy of Cinema Guild)

In the fall of 1976, after a year of political activism, the Sociobiology Study Group began losing its momentum.^30^ The flyers advertising *Sociobiology: Doing What Comes Naturally* revived their commitment (Fig. 2).^31^ It must have felt like déjà vu: the flyer announced once again the advent of a new discipline of sociobiology “with revolutionary implications for the disciplines biology, anthropology, sociology, and psychology.” It once again quoted Wilson stating that analyzing the social organization of lower animals will help explain the evolution of human social behavior. And it once again addressed a general audience of “Senior High, College Adult” students. The flyer confirmed the suspicions that Freda Salzman so concisely summarized: sociobiology would be used to indoctrinate the youth, undermine the social sciences, and influence the American public toward reactionary policies.

Next to the publication of *Sociobiology* itself, this film of little over 20 min sparked the most virulent attacks and provoked intense engagement. The critics found fault with the sensational claims of the flyer and the ambition exemplified in the film. Beckwith recalls: “Trivers epitomized the ambition of the sociobiologists … ‘that we should start setting up a physical and social world which matches [biological] tendencies.’ Such statements reflected the belief of many sociobiologists that their science would ultimately be important for the development of social policy” (Beckwith 2002, p. 144). SftP moved quickly, bought the film for the group soon after it was advertised,^32^ and organized a public screening by mid-December 1976.

Predictably, SftP did not like it one bit. By 31 January 1977, Tedd Judd had prepared a draft of a review entitled *Sociobiology: Naturizing What We Do*, which summed up their position: “The film … is riddled with blatant sexist and racist political messages” read the first sentence, and what followed was a detailed summary and critique of the film.^33^ Objections were, in particular, made to the evidentiary basis of DeVore’s statements regarding primate life and reproductive control, the political bias of Trivers’s statements on warfare, rape, and sexual behavior, and the disciplinary hopes laid out by Wilson. Especially contested is Trivers’s statement that the evolutionary analysis of behavior is an “extremely powerful concept that has lain dormant since Darwin,” as SftP had emphasized for over a year now that sociobiology was the latest iteration of biological determinism in one historical line with Social Darwinism, the eugenics movement and ethology: “Sociobiology is simply the latest reappearance of these same tired arguments and the phallacies^34^ underlying it are apparent in this film.”^35^ The draft ended with an appeal: “Because this film so obviously perverts the scientific educational purposes for which it was made and acts instead as a piece of sexist, racist propaganda, we urge that it be removed from circulation.”^36^

**Fig. 3:**
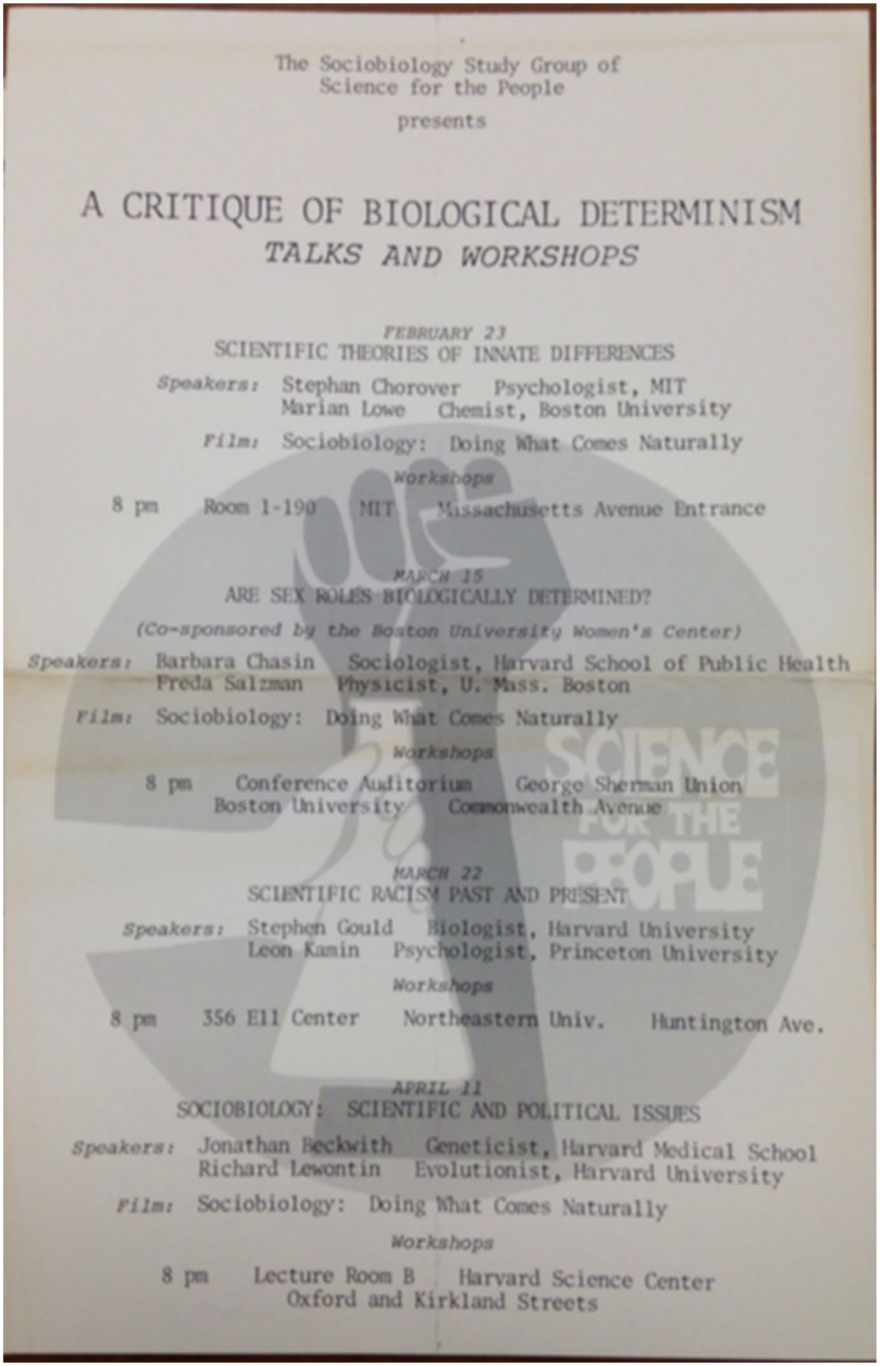
Flyer advertising the *A Critique of Biological Determinism. Talks and Workshops* series, taking place in the spring of 1977 in the Boston area. The series was organized by the Sociobiology Study Group of Science for the People and featured the most prominent critics of sociobiology, Jonathan Beckwith, Stephen Jay Gould, Stephan Chorover, Leon Kamin, Richard Lewontin, and Marian Lowe. Barbara Chasin and Freda Salzman held the lecture, “Are Sex Roles Biologically Determined?” (co-sponsored by the Boston University’s Women’s Center) before showing and holding a workshop on *Sociobiology. Doing What Comes Naturally*. (The Sociobiology Study Group of SftP, “A Critique of Biological Determinism: Talks and Workshops,” flyer, spring 1977, Box 185, Edward O. Wilson Papers, Manuscript Division, Library of Congress)

However, over the spring of 1977, SftP itself organized multiple showings of the film. Worried that it might prove to have mass appeal and proliferate its message without critique and comment, SftP quickly adopted another strategy than a call for removal: providing context. Building on the traditions of collective action that some members employed since the civil rights struggle in the 1960s (Sheldon 2013, pp. 105–107; Sheldon 2014, pp.70–77), SftP not only hosted a weekly study group but organized workshops and lectures. They used these methods to build a series of lectures on sociobiology that began using the film as a visually engaging representation of sociobiology. This took place most prominently at the universities in the Boston area and at the University of Michigan in Ann Arbor but likely spread to other campuses.^37^ The Boston showings took place between February and April 1977 as part of “A Critique of Biological Determinism: Talks and Workshops” (Fig. 3).^38^ The film was shown after the talks “Scientific Theories of Innate Differences”^39^ at MIT, “Are Sex Roles Biologically Determined?”^40^ at Boston University, and “Sociobiology: Scientific and Political Issues”^41^ at Harvard. Flyers for these events show that the film was used as an “illustration” of the problems associated with sociobiology and that it “typifie[d] the attempts to attribute the worst aspects of our society, e.g., aggression, sexism, etc., to part of inherited ‘human nature.’”.^42^

Some showings were supplemented by a workshop on the film. A worksheet entitled “Questions for the workshop on the film”^43^ first asks about the emotional impact, offering a multiple-choice answer: “Does the film make you feel excited about a ‘new’ science? reassured about our society? outraged by its sexism and racism?” One assumes the expected answer was the last. The worksheet then walks the participant through the steps of critically analyzing the film: It identifies the messages the film conveys,^44^ then suggests it does so by creating “links to popular ideas,”^45^ including some “benign implications,”^46^ and giving them “scientific clothing.”^47^ After asking the participant to consider for themselves the scientific basis for the statements and the historical line into which sociobiology falls, it concludes that it presents the “biological determinism position.”^48^ The conclusion then guides the participant to assess the social impact of this position by asking who benefits most from it and who supports research that bolsters it.^49^ The worksheet ends with item 7, “Action—What can we do?” The worksheet moved from analysis to action, thereby effectively turning participants into activists and generating more support for public outreach critical of sociobiology, such as protests, letter-writing campaigns to journals and magazines, and more public lectures and events.

The minutes of SftP meeting after one of these workshops suggest that the extensive analytical framing of the film might have even been too obvious; the feedback given was that the “film does not need lengthy introductory analysis” and that despite the topic of the workshop “the show gave the appearance of being run by men, with women being put up to speak.”^50^ Still, over 250 people attended the showing, and the workshop was considered a success. After spring 1977, the frequency of showings in the Boston area is less well documented and might have dwindled as the summer issue of SftP Magazine advertised the availability of “Sociobiology Speakers” and “in some cases, a showing of the film.”^51^

These showings, as well as the workshops at Harvard, demonstrate that SftP were very effective at approaching, shaping, and convincing specific audiences of the biological determinist dangers posed by sociobiology. The focus on getting the context of historical narrative and political implications across, as well as targeting specific audiences in using the film reflected their overarching strategy during the sociobiology debate: in addition to putting Wilson on the defensive in the public sphere, SftP built their own critical audience in the New Left and collective action-based academic circles. While Wilson used the image of sociobiology as a revolutionary and relevant science of human nature to promote his new discipline, SftP successfully painted a picture of yet another reactionary pseudo-science with political implications of racism and sexism.

## Sociobiology Misrepresented: Denouncement of Sociobiology: Doing What Comes Naturally

The effective use of *Naturally* by SftP to build support for their critique of sociobiology confronted Wilson, DeVore, and Trivers with a challenge: stopping further showings of the film. While SftP publicized it as a product of sociobiological reasoning, the sociobiologists disagreed and felt that their field was grossly misrepresented. The immediate impact and long-lasting legacy of the film are, therefore, deeply connected to the question Austrian journalist Georg Breuer posed in 1983: “But is this film merely misrepresenting sociobiology or does it depict, in a crude and simplified form, ideas that are indeed in the heads of some sociobiologists?” (Breuer 1982, pp. 252–253). This question was answered very differently depending on who one asks: SftP claimed that the film is entirely representative and the outrage it caused is a direct result of the statements of the sociobiologists. The sociobiologists argued their position that only a double re-contextualization caused the outrage. The first re-contextualization was due to the production elements that Hobel-Leiterman added, the sound effects, music, narration, and stock footage, and the second re-contextualization occurred because SftP showed the film in the context of workshops with introductory analysis beforehand and leading questions afterward, priming the audience to find their statements offensive. Due to these re-contextualizations, they were desperate to distance themselves from the film in any way possible after seeing it for the first time in December 1976, using anything from legal challenges to public denouncements.

Some of their colleagues agreed with the sociobiologist’s reasoning. Edward R. Buchler, professor of zoology at the University of Maryland wrote: “The producers chose to retain those statements … that were largely speculative and then to make even further unwarranted extrapolations from these … . Even though Dr. Wilson’s comments were conservative and carefully qualified, inferences of the narration were totally inappropriate” (Buchler 1979, p. 109). Other members of the biological community were torn; while they acknowledged that the production added significantly to the misrepresentation, they also attributed some responsibility to the sociobiologists and called for more careful consideration of such statements. Richard Alexander, professor of evolutionary biology at the University of Michigan and himself pursuing evolutionary approaches in human behavior at the time, warned Wilson in a letter that “all of us need to take very seriously the fact that such things can be done with what seems to us innocent and reasonable remarks and statements, and that they may even be done in good faith—that is, under the belief that they represent an accurate interpretation of the facts and theory involved in the current controversy. It’s food for thought.”^52^

**Fig. 4:**
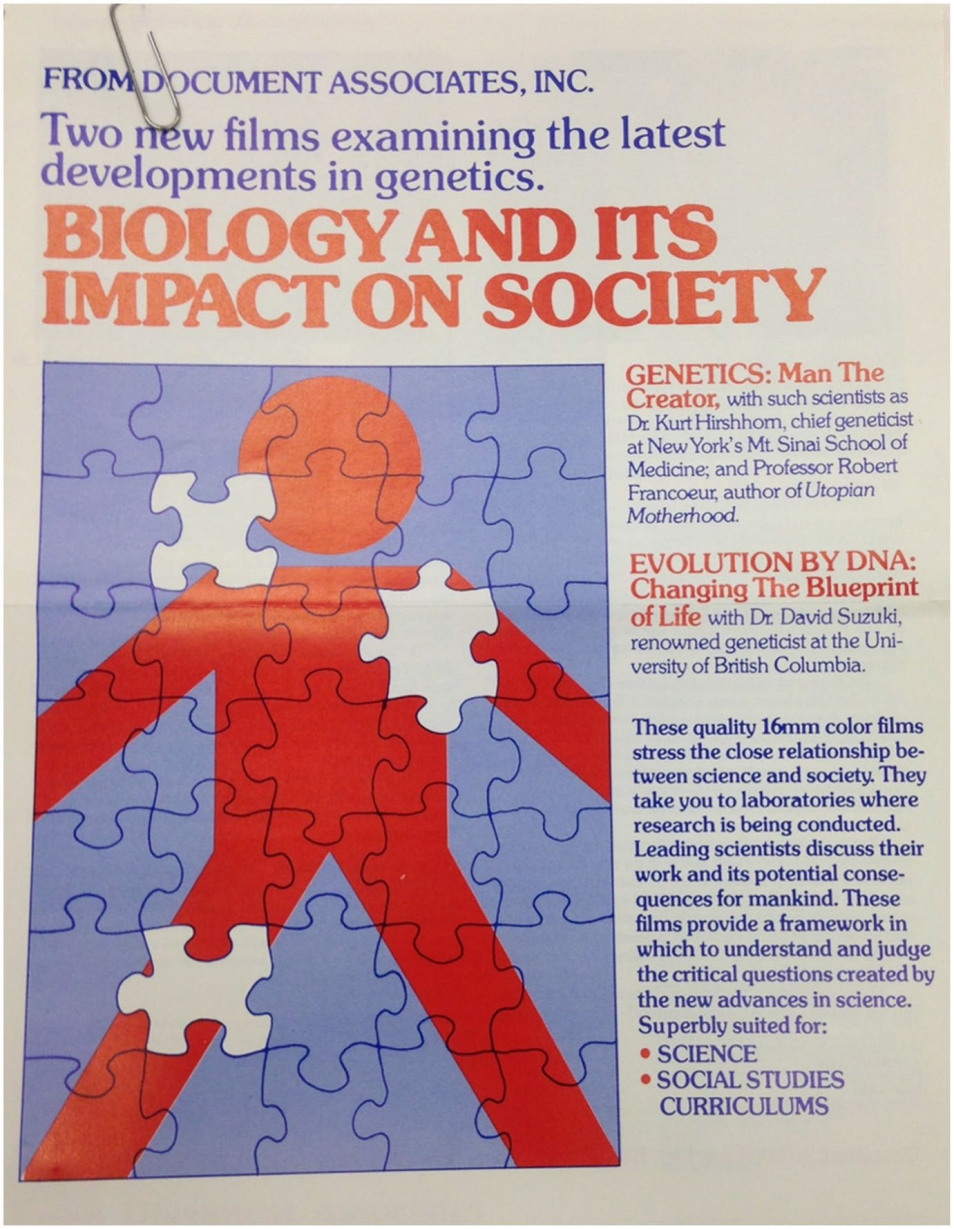
Front cover of a leaflet advertising the addition of “Genetics: Man The Creator,” and “Evolution by DNA: Changing The Blueprint of Life” to the *Biology and Its Impact on Society* documentary series. Other entries include “A Fat-Free Society: The Girth Control Controversy” covering the Atkins’ Diet, “Learn to Live With Stress: Programming the Body for Health” on “stress as a killing disease,” and “To Make Man Into Superman” on the cryonic movement (all Hobel-Leiterman Productions, 1976). *Sociobiology. Doing What Comes Naturally* is not included in the series, pointing to the film’s legal issues. (Document Associates, “Biology and its Impact on Society,” advertising leaflet, Box 186, Edward O. Wilson Papers, Manuscript Division, Library of Congress. Courtesy of Cinema Guild)

The sociobiologists took up legal measures to fight what they deemed a misrepresentation of sociobiology in the film, claiming that Hobel-Leiterman had effectively distorted their statements due to the overwhelming impact of the production elements (the narration, sound effects, music, and stock footage). Their initial strategy, therefore, attempted to get the film removed from circulation as they felt that Hobel-Leiterman went past their legal rights to use the interviews. They later even entertained suspicions that they were deliberately duped into giving consent to a commercial film and stressed the lack of a script at the time of the interviews. Their first step was a letter to the production company written on their behalf by the EDC. The letter ends by asking the production firm to “recall all prints of the *Sociobiology* film and remove our footage from your A and B rolls and internegative of the film.”^53^ Ironically, then, there was a brief moment in time in early 1977 when both the sociobiologists and SftP agreed that the film should best be removed from circulation. This was not Leiterman’s view: his response defends the film against any charge of willfully misleading the public as to the content of sociobiology, claiming they had “received enthusiastic responses on the film from both the scientific community and from educators” and hoping for understanding that “any attempt to render the entire subject of Sociobiology in 20 min of film must necessarily abridge the concepts treated, and perhaps be seen as superficial … . This film was very much a labour of love for our people who made it, and its very modest circulation over the years has not even repaid the costs of production.”^54^ While the argument of a misuse of the interviews was unsuccessful in getting the film removed from circulation, additional copyright infringement claims by the EDC against Hobel-Leiterman Productions forced them to recut the film to avoid further legal action. By 1978, the production company distributed a new version without the copyrighted material and with toned-down visuals and narration (Fig. 4).^55^

When the workshops started in February 1977, it was clear that the sociobiologists’ previous efforts were not enough to dissuade SftP from further showing the film, so they prepared a public denouncement of both the film and its showings. In a letter dated 30 March 1977 to the local student newspaper, *The Harvard Crimson*, they elaborated the points they had already made in the letter to Hobel-Leiterman.

We were chagrined to discover that the content of our interviews had been overwhelmed by a tasteless, sensationalized production that caricatured the field of sociobiology. Since we have taken care to present our views elsewhere in a spirit of responsible scientific inquiry, it is especially galling to see these views parodied by the material interpolated between the interviews: a naive and misleading narration, footage chosen more to shock than inform, and a hard-rock musical background that leaves little doubt that such impressions are intended. The result is a pastiche that seriously distorts our views.^56^

Now, in addition, they also charged SftP with the illegitimate use of the film after they had already denounced the film after its first showing: “Despite the fact that we explained the above sequence of events to the audience at the December [1976] screening of *Naturally* and on various occasions since that time, speakers on behalf of SftP continue to show the film at public meetings. We deplore the vulgar misrepresentation of sociobiology by this film; we equally deplore the misrepresentation of the field by those who use this discredited film to imply that it represents an accurate statement of our ideas.”^57^

With this letter, the sociobiologists publicly established the two-pronged approach of pointing to the misleading production and misrepresentation by SftP as a way of distancing themselves from the film. However, SftP was not impressed by this argument; a *Crimson* article from 11 April 1977 covering a planned showing quoted Lewontin saying the film is “certainly shocking and vulgar, [but] if I thought it was inaccurate I wouldn’t show it” and in the same article Beckwith stated: “The film is basically a vulgarization of what they say … but I don’t think it’s inconsistent with their statements. I think they are naive for not anticipating that this is what happens to theories like those they propose” (Printz 1977, p. 4). In the end, despite sociobiologists’ efforts the film was shown as planned and even the article itself ends with the announcement: “Sociobiology: Doin’ What Comes Naturally, is showing tonight at 8 p.m. in Science Center B.”

An especially embarrassing showing occurred at a lecture by evolutionary theorist William Hamilton at the University of Michigan in October 1977. The local SftP chapter, the second-most active in their anti-sociobiology activism after Boston, protested the lecture due to Hamilton’s theory of inclusive fitness, which was being perceived as part of sociobiology. Evolutionary biologist Richard Alexander, the professor who had invited Hamilton to give the lecture, reported in a letter to Wilson, “the method they finally selected is extremely effective: They will simply show … ‘Naturally’ … . I am not aware that any evolutionary biologists have ever used the film for any purpose other than ridicule or amusement.”^58^ Alexander took Wilson to task for his involvement with the film: “This letter is an expression of resentment that, for whatever imagined or real profit, you could have participated in producing such an inferior, misleading, sensationalized device.”^59^ However, Alexander was subsequently informed about the public letter by the sociobiologists, reprinted in the October 1977 issue of *Anthropology Newsletter.*^60^ Alexander then wrote a second letter chastising the lack of effectiveness of their public distancing strategy because *Anthropology Newsletter* was “not available to evolutionary biologists.”^61^ He also rejected Wilson’s other defense: “Come on, Ed, you’ve got to be kidding! Surely you haven’t got the guts to send me a 30 March copy of the *Harvard Crimson* to prove that you and your colleagues really did publicize your dissociation with that silly film long enough ago to make my letter ‘behind the times.’ … In view of the manner in which you guys manage publicity when you’re sticking your horns out, it’s amazing how slowly and circuitously things move when you’re pulling them in.”^62^

Maybe this stern admonishment by a colleague pushed the sociobiologists to broaden their strategy as denouncements of the film were printed in more newsletters in the following years.^63^ The British magazine *New Scientist* also covered the story. DeVore made a point to contrast the dubious methods of Hobel-Leiterman with the paragon of journalistic integrity, the BBC: “‘My previous experience with the BBC had lulled me into a sense of security—but we came unstuck this time,’ DeVore now remarks bitterly.” Then the familiar two-pronged approach is employed: Firstly, the dubious production: “The three of us saw the film with some shock … not for what we said in the interviews—much of which we still stand by—but because they were interspersed with shots of heavy petting, flaming napalm, and baboons fighting, all against a background of a hard-rock beat. The overall effect is a naive biological reductionism.” Secondly, the equally dubious methods of SftP and their continued showings “as a way of attacking sociobiology” despite the sociobiologists having already denounced the film (Lewin 1977, p. 711).

Despite their best attempts to publicly denounce the film, the sociobiologists appeared to be always one step behind SftP, doing damage control rather than controlling the narrative surrounding the film. This is exemplified further in SftP often getting in the last word. For instance, the Ann Arbor’s chapter replied to DeVore in the *Anthropology Newsletter* in December 1977 with the claim that merely denouncing the film was not enough, as it was not just the production that contained dangerous messages: “We believe that [their] statements discredit sociobiology just as much as the narration, soundtrack and footage of the film … . Indeed [they] show beyond any doubt that the vulgarization of Darwinism is due at least in part to two of the country’s most respected sociobiologists” (Boucher et al. 1977, pp. 19–20).

When Tedd Judd’s review was finally published after a year of activist peer-review in January 1978,^64^ little had changed compared to one year earlier. Despite a recent legal agreement with Hobel-Leiterman to recall the film, SftP had controlled the film’s impact. SftP was happy with this achievement and even attributed the denouncements of the film to their actions.^65^ Judd concludes that it continues to be legitimate “to use the film as an illustration of the political nature of these statements and as an example of the ease and speed with which the speculations of sociobiologists are picked up and used in support of political positions. We join these scientists in hoping that the film will be recalled and no longer used in the indoctrination of high school students (Judd 1978, p.19)." In fact, at the time of publication, the film had already been recalled in December 1977 due to copyright issues. However, SftP continued its showings of the original version for years—it had become too effective a public outreach tool to set aside by then.^66^

## Sociobiology: Doing What Comes Naturally’s Polarized Legacy: Whose “Cinematic Catastrophe” was It?

The final words of Judd’s review claimed that the film itself, without contextualization, was likely to indoctrinate naïve youngsters. But was the film actually as dangerous as feared? It seems that members of SftP considered the film more influential than it might have been.^67^ Initially, Leiterman maintained that the film sold so few copies it did not even break even with production costs.^68^ However, in 1980 SftP member Jan Fritz wrote that sales and rentals were good, according to the distributor. It is unclear whether Leiterman’s statement was a strategy to convince the EDC to leave the film alone or whether the controversy itself helped the film’s circulation.

What about its impact on the audience? It is hard to discern what general audiences made of it as most reviews were written by either members of SftP or close associates of sociobiologists. One reviewer wrote that students reacted negatively and that “for the naïve, it is a potentially dangerous film;” it was “technically mediocre, sensationalistic, superficial and misleading” (Buchler 1979, p. 109). This impression was shared by Fritz, who found that “university students … thought it was so bad that it was funny. The students were concerned, however, that the film might be effective with an uninformed audience” (Fritz 1979, p. 65). Jeffrey Kurland, a graduate student of DeVore, agreed that the film was a “cinematographic catastrophe” and joked: “A sucker for adaptational explanations, I succumb to temptation. Naturally was actually put together by SftP in order to step up campus unrest and thus hasten the coming of the Revolution. In return for a share of the royalties, DeVore, Trivers, and Wilson were actually in on this cinematic Piltdown” (Kurland 1983, p. 267). Perhaps this indicates that the film was not ideally suited to the task of indoctrinating viewers into sociobiology.

Kurland’s joke about the film being a false flag operation reflected a rumor that “suggested that the film might have been made by the opponents of sociobiology as an attempt to discredit it” (Boucher et al. 1977, pp. 19–20). The Ann Arbor SftP group denied this firmly: “As viewers of the film will realize, such a scenario is laughable” (Boucher 1977, p. 20). However, the intimate connection between the film and SftP-sponsored showings led to the rumor spreading to even *BioScience* editor John Behnke: “If you haven’t seen the film distributed by the SftP group, you have missed what I consider the lowest point to which supposedly reputable scientists have sunk. They got hold of a taped interview with Wilson and doctored it by dubbing in ridiculous, irrelevant pieces and cutting statements to leave pieces out of context—all designed to make Wilson look ludicrous.”^69^ These false allegations are illustrative of the already paranoid and polemic atmosphere of the debate, and how *Naturally* only added oil to the fire. The immediate impact of *Naturally* was to reignite the sociobiology controversy and intensify the impression of both parties that the other had violated basic tenets of respectable behavior: The critics felt that the sociobiologists had once again bypassed the proper channels of the scientific community in order to popularize sociobiology in front of a naïve audience of students and laypeople; the sociobiologists felt that their critics in SftP had once again broken the rules of public debate in disregarding their denouncement of the film. The film made each side look worse in the eyes of the other side.

The passage of time strengthened these assessments. References to *Naturally* in critical articles show that it was taken to stand *pars-pro-toto* for the entire discipline of sociobiology itself and representative of the ideas of its founders. While earlier articles reviewed the film itself, later articles used the film to make more sweeping points about sociobiology. Often, they did not go into details about the film or the flaws of sociobiology, but the mere mention of it conjured up enough images with which to work. This change in discussion of the film occurred between the late 1970s and the mid-1980s. Especially, feminist academics prominently used the film as an illustration of sociobiology’s flaws (Nuyen 1985, p. 179; Lowe 1978, pp. 118–125; Chasin 1977. See also Tang-Martinez 1997, pp. 116–150). The 1970s assessment of the film as dangerous—“Exciting and endearing clips move so rapidly that the eye is engaged and distracted as the voice-over slips in the message of the genetically fixed ‘laws’ of human nature”—and the call to contextualize it—“This film cannot be ignored or stopped: rather, it must be exposed as a piece of political propaganda” (Marlowe 1983, p. 204)—were widespread. The interview sections about sex-determined behavior became a reference point as “pseudoscientific nonsense” (Marlowe 1983, p. 204) for feminist critiques of sociobiology. By 1985, the film was listed as an excellent “pro-sociobiology” visual representation without any further qualifications (Rosser 1985, p. 212).

Using the film to criticize sociobiology was not limited to feminist scholarship but extended to the history, sociology, and philosophy of science. In 1984, *Naturally* was shown at “The Sociobiology Debate: A Retrospective,”^70^ an event sponsored by the Harvard Radcliffe Society for the History and Philosophy of Science with Lewontin, Gould and other members of SftP presumably once again as representative of sociobiology. Eventually, even the line between the film being representative of sociobiology and being attributed to sociobiologists itself becomes blurry: In 2001, Michael Fischer, professor of anthropology and science and technology studies, wrote, “Many of us remember an astonishing film the sociobiologists *helped produce* in the 1970s …” (Fisher 2001, p. 13, emphasis added). While the sociobiologists did give interviews, it is a stretch to claim that they helped produce the film or to refer to *Naturally* as “their film,” as philosopher John Troyer did in an article in 2000 (Troyer 2000, p. 64). *Naturally* to these scholars was a resource to promote sociobiology, representative of its content and ambitions, or even created and approved by E.O. Wilson himself.

However, others, often evolutionary-minded social scientists, remembered the same film as a resource to attack and discredit sociobiology. The use of *Naturally* as an instrument of leftist critique was so successful that the film itself came to be misremembered as an attack piece by SftP. My interview with John Tooby exemplified this. When asked about the film, Tooby misremembered the film as “highly critical” of sociobiology and not including “anybody I actually recognized as a scientist.” Then a memory came back to him: “I remember Bob Trivers said … . He was in the film!” At first, Tooby had retrospectively inserted the critical contextualization of the SftP showings and the critical academic analysis into the film itself. But when he recalled Trivers’ participation in the film, he quickly adjusted his recollection to include that the film must have taken him out of context: “It had three sentences by somebody and then a primate and then three other sentences by somebody else and then some rock music, but it’s things that are so denuded of context … . But Bob said: I stand by everything I said in that film.” Tooby’s answer also employs the distinction between sociobiologist’s statements and how the production of the film re-contextualized their interview statements. This shows that the sociobiologists enjoyed some success with their strategy to distance themselves from the film and there remained a lingering sense that the film was a misrepresentation of sociobiology.^71^  In fact, the suspicions, rumors, and allegations regarding the production and distribution of the film proved to have staying power to this day, as a recent Twitter post claims, “[this] brings back memories of Naturally, a 1976 doc released *under the auspice* of the Sociobiology Study Group (feat. Lewontin), in which heavily edited bits of interviews with Trivers, DeVore, and Wilson were interspersed with shots of napalm and baboon fights.”^72^

For both sides of the sociobiology debate, *Naturally* took on a symbolic meaning, encapsulating the content and methods the other party used to convince and corrupt audiences. The film itself is overlayered by this symbolic function; the content or creators become vague details that could be made to fit respective purposes. In some circles, the film became a symbol for the sexist outlook of sociobiology, in others for the ruthless methods of SftP. Not surprisingly, these circles display a strong correlation with disciplinary boundaries as scholars of the evolutionary social sciences that followed in sociobiology’s footsteps remember it for its use as an attack on sociobiology.^73^ In contrast, disciplines with connections to the critical spirit of SftP, such as history and philosophy of science or Science and Technology Studies, are more likely to know it as a problematic product of sociobiological thinking.^74^

This mirrors the polarized legacy of sociobiology in academia. One side was attacking it, the other was defensive of the burgeoning discipline and apologetic of its flaws. Since neither side could be convinced by the other, this resulted in two diverging historical narratives of the sociobiology debate. Myers summarized them: “Critics tend to act as if sociobiology were publicly discredited, whereas sociobiologists tend to act as if the political criticism were a thing of the past (neither of which views is supported by a survey of articles published in the last few years)” (Myers 1990, p. 245). Almost 50 years after the publication of *Sociobiology. The New Synthesis* one can extend this assessment to disciplinary developments: Even though sociobiology failed to establish itself as a discipline in its own right, it still left its mark as an incomplete and flawed, but important forerunner to the evolutionary social sciences that in turn attract similar criticisms as sociobiology did in the 1970s.

## Footnotes

1 “Freda’s Black Rose Lecture, 11/18/1977: Scientific Sexism: Freud to Sociobiology.” Papers of Freda Friedman Salzman, 1927–1981, 83-M100–83-M173: T-155, T-155.7. Schlesinger Library, Radcliffe Institute, https://nrs.harvard.edu/urn-3:RAD.SCHL:39294838 (accessed 7 December 2022).

2 There is no published account of Freda Salzman’s life, but the Schlesinger Library, Radcliffe Institute at Harvard Library holds her papers, https://hollisarchives.lib.harvard.edu/repositories/8/resources/9171. Additional information from a website her husband, George Salzman, has dedicated to her memory, http://site.www.umb.edu/faculty/salzman_g/Freda/index.htm.

3 The Black Rose Collective was an Anarchist group in the Boston area, which intermittently published a magazine, circulated leaflets, and started a lectures series, “The Black Rose Lectures,” in 1975 which pursued “the introduction of new approaches and ideas to the issues of our age, and a re-examination of past understandings from a revelatory or critical perspective.” (Hess 1979, pp. 2–6).

4 *Sociobiology: Doing What Comes Naturally*, directed by Richard Loth (1976/1978). Some of the background of the film as well as its reception has recently been covered in Milam (2018, pp. 250–256).

5 Some of the interviewees were involved in developing an educational curriculum for high school use, entitled *Man: A Course of Study,* that also relied heavily on the moving image. Irven DeVore contributed to this curriculum by providing footage of baboon social life and Robert Trivers wrote booklets and other materials. For more on the curriculum, see Milam (2013). For an exploration of multivalent film in the case of British ethology, see Kempton (2023). For more on multiple uses of footage in different contexts, see a recent special issue “Reusing Research Film and the Institute for Scientific Film (Sattelmacher, Schulze and Waltenspül 2021). The duality between science and entertainment in non-fiction science films has been noted by scholars of film such as Oliver Gaycken who posits that popular scientific moving images construct knowledge, but also enable circulation beyond their original context for artistic and academic purposes, in a “transit between visual cultures of scientific visualization and entertainment cinema” (Gaycken 2015, p. 3).

6 John Tooby (Professor, Department of Anthropology, University of California, Santa Barbara and Co-Director, Center for Evolutionary Psychology), interview by author, Santa Barbara, CA, October 28, 2018.

7 For another example of close reading of scientific films, see Gaycken (2002, pp. 362–370).

8 I pursued several avenues of acquiring the original film: (1) I looked for library catalogs that indicated that the original 24-min version was in possession. Likely sources were Harvard, where most members of the Boston SftP chapter were based, and Pennsylvania State, where Jeffrey Kurland, a reviewer of the original film was based. However, both libraries could not provide the film (PennState had the recut version, Harvard did not have any version). (2) WorldCat has nine listings for the film. Despite being dated 1976, requests at the libraries revealed that they were in possession of the recut 1978 version because there was no footage of Anubis baboons, (3) The Canadian Archive for Educational, Sponsored and Industrial film was searched and its curator contacted. (4) The legal successor to Hobel-Leiterman Productions, Cinema Guild, was contacted about a copy of the original release or additional information about the film, without success. (5) After archival sources showed that Stephan Chorover, professor of psychology and active member of Science for the People, originally purchased the film for the group. I contacted his next of kin (Chorover passed away in 2015). I also contacted next of kin of Jeffrey Kurland (Kurland passed away in 2018) and the founder of the production company Hobel-Leiterman productions. All without response or success.

9 For another example of the reconstruction of a controversial lost film using its reception, see Wazeck 2010, pp. 163–179.

10 Segerstråle was a Ph.D. student at Harvard at the time of the debate. She conducted extensive interviews, which form the basis for her Ph.D. thesis (Segerstråle 1983). The later publication as a monograph was additionally informed by the so-called Science Wars of the 1990s.

11 In 1986, Wilson read and endorsed Segerstråle’s published account of the debate (Segerstråle 1986) as “basically right” in their correspondence and used it in his courses on human sociobiology. When writing his own autobiography in 1994, he read her unpublished thesis (Segerstråle 1983) for the first time in its entirety. In a letter he asked her to read the relevant chapters on the sociobiology debate, complimented the thesis as a “superb piece of scholarship and insightful thinking” and encouraged her to publish it, E.O. Wilson to Ullica Segerstråle, March 15, 1994, Box 151, Edward O. Wilson Papers, Manuscript Division, Library of Congress, Washington D.C. (hereafter cited as EOW Papers). Similarly, he asked Stephen Jay Gould and Richard Lewontin to read the chapters covering the sociobiology debate, E.O. Wilson to Stephen Jay Gould, March 30, 1994, Box 79, EOW Papers; E.O. Wilson to Richard Lewontin, March 30, 1994, Box 106, EOW Papers. Gould agreed to read the chapter and offered some notes, E.O. Wilson to Stephen Jay Gould, April 19, 1994, Box 79 EOW Papers. Lewontin declined to be involved with the reasoning that the “disagreements about sociobiology are so fundamental and so broad” and that there is a “widespread misapprehension that our disagreements are personal rather than scientific in nature,” Richard Lewontin to E.O. Wilson, May 18, 1994, Box 106, EOW Papers.

12 Richard Lewontin to E.O. Wilson, October 28, 1975, Box 106, EOW Papers.

13 In his autobiography, Wilson recounted the perceived threat by molecular biology, represented by James Watson, to evolutionary biology and the ensuing inner-departmental struggle at Harvard under the title “The Molecular Wars” (Wilson 1994, Chapter 12). For more on the institutional and disciplinary background of sociobiology, see Sleigh 2007, Chapter 9.

14 After the public attacks by Science for the People, Wilson’s promotion turned into a defense of himself and sociobiology. He published a book-length defense of sociobiology entitled “On Human Nature” (Wilson 1978), even winning a Pulitzer Prize for General Nonfiction in 1979.

15 Science for the People was a left-wing group which emerged in the late 1960s to oppose militaristic influences in the sciences, the US military involvement in Vietnam, to promote progressive domestic politics and to demand an end to sex- and race-based discrimination. During the early 1970s the Boston chapter of the organization developed a special focus on issues relating to the social uses of biological reasoning, such as explaining criminal behavior with genetic explanations or claiming the existence of differences in IQ between races (Moore 2008, pp. 176–184). For more on the history of Science for the People see: Moore (2008), Moore (1996), Agar (2008), Wisnioski (2003). Schmalzer et al. (2018) provides a collection of documents of Science for the People documents with brief introductions to the historical background.

16 The Sociobiology Study Group was organized as a consensual nonhierarchical group with fluctuating membership oscillating between being a study and an action group. Throughout the controversy, especially Richard Lewontin and Stephen Jay Gould became the faces of this group, even though at least Lewontin was unhappy with this development. He publicly stressed the nonhierarchical nature of the group and the limited influence prominent members had. The Sociobiology Study Group refers to itself mostly as *Sociobiology Study Group of Science for the People*, but in the context of the debate the moniker *Science for the People* became more prominent. Therefore, I refer to the Sociobiology Study Group by Science for the People in the following as *Science for the People*, abbreviated as SftP.

17 Sociobiology Study Group minutes, August 7, 1975, Box 888, Folder 1, Stephen Jay Gould Papers, M1437, Dept. of Special Collections, Stanford University Libraries, Stanford, Calif. (hereafter cited as SJG Papers).

18 The sixteen members of the group signed the letter alphabetically, resulting in Elizabeth Allen, a medicine student at Brandeis University, being named as the author in conventional citations. This was done to underscore the non-hierarchical nature of the group, but the principal author of the letter was Stephen Jay Gould, who prepared a draft that was then extensively reworked by the group. Draft of New York Review of Books Letter, Box 888, Folder 5, SJG Papers.

19 The production company Hobel-Leiterman Productions and their distribution company Document Associates were based in New York and Toronto. Douglas Leiterman had already achieved success in Canada with the public affairs show “This Hour Has Seven Days” which aired from 1964 to 1966 and later founded Hobel-Leiterman Productions with Philip Hobel. Hobel and Leiterman were successful filmmakers with even an Oscar nomination for Hobel in 1984 and Leiterman being considered an important figure in Canadian film. “Producers Phil Hobel and Doug Leiterman had a happy knack for producing slick and often award-winning documentary series with a maximum of presentational flair. In their hands, even the most trivial piece of information became a triumph of style over substance” (Wedge 2002).

20 Wikipedia has more details on the controversial opening credits to the TV series: “The series had a unique opening scene featuring a nude blonde girl, seen from behind, walking from the beach into the surf until she disappears under the surface to swim underwater. This opening title had various clips of different thought provoking scenes superimposed over the model, of people and new technologies of the time, so as to distract from the nudity which was provocative for early 1970s Canadian television.” Wikipedia’s entry on “Here Come the Seventies”, last edited 26 March 26 2022, https://en.wikipedia.org/wiki/Here_Come_the_Seventies, last accessed 23 March 2023. The reader can confirm the veracity of this description on YouTube: “Here Come The 70 s opening,” YouTube video, 00:43, uploaded by Tomogram, 9 January 2015, https://www.youtube.com/watch?v=37z6eAp3D4A (accessed 23 March 2023). As the general tone of the production becomes an issue in later discussions of the film, it is worth noting that the tone of *Naturally* does not seem to differ from the general tone of the series.

21 This is also the title of the last chapter of his previous book *Insect Societies* (Wilson 1971b). In the same year he also laid out his ideas in the last paragraph of an article in *Science* (Wilson 1971c).

22 The term “sociobiologists” is used throughout the article to refer to E.O. Wilson, Irven DeVore, and Robert Trivers collectively in the context of the film. Both DeVore and Trivers have either completely ceased to refer to themselves as sociobiologists or do not consistently refer to themselves as sociobiologists.

23 “Hamilton, The Father, Bob Trivers, the Son and George C. Williams, The Holy Ghost (no one had met him yet and he was not known in fields like primatology, anthropology, sociology, etc.).” Jeffrey Kurland quoted in Grodwohl (2017, p. 30); see also Grodwohl (2019). For more on Trivers’ contributions, see Milam (2010, pp. 138–159).

24 Both releases were produced by Hobel-Leiterman productions and distributed by Document Associates.

25 All of the following reviews provide direct quotes or descriptions of specific scenes: Tedd Judd, Draft of Review, January 31, 1977, Box 888, Folder 1, SJG Papers; Judd (1978, pp. 16–19); Kurland (1983, p. 267); Fritz (1980, p. 99).

26 Judd, Draft of Review, p. 1.

27 Judd, Draft of Review, p. 1.

28 Judd, Draft of Review, p. 5.

29 Irven DeVore, Robert L. Trivers, and E. O. Wilson to *Harvard Crimson*, March 30, 1977, Box 186, EOW Papers.

30 “*Collective Spirit:* Jon … had been disturbed by a general reluctance to volunteer for shitwork and even for more interesting tasks but felt that the forums [in February 1977] had inspired fairly general enthusiasm and involvement.” Sociobiology Study Group minutes, March 1, 1977, Box 888, Folder 1, SJG Papers.

31 Document Associates, Advertising flyer for *Naturally*, Box 186, EOW Papers.

32 As shown by a letter Stephen Jay Gould send to the distributor: S. J. Gould to Ernest Schimizzi, December 10, 1976, Box 694, Folder 1, SJG Papers.

33 Judd, Draft of Review, pp. 1–6.

34 The author warns at the top of the first page that the draft includes “intentional jokes to be removed from the next draft.” Judd, Draft of Review, p. 1.

35 Judd, Draft of Review, p. 4.

36 Judd, Draft of Review, p. 6.

37 The film was also shown to more specific audiences: In one instance, on March 25, 1977, an open forum discussing Sociobiology was organized for the Northeastern Anthropological Association at Northeastern University in Boston with Stephen Jay Gould, Richard Lewontin, and Stephan Chorover participating. This points to the particular importance of reaching disciplinary publics within the social sciences, especially anthropology. Film Symposium Announcement, March 25, 1977, Box 888, Folder 1, SJG Papers.

38 The Sociobiology Study Group of SftP, “A Critique of Biological Determinism,” flyer, spring 1977, Box 888, Folder 1, SJG Papers; The Sociobiology Study Group of SftP, “A Critique of Biological Determinism,” flyer, spring 1977, Box 185, EOW Papers.

39 Stephan Chorover and Marian Lowe on February 23, 1977, at MIT.

40 Barbara Chasin and Freda Salzman on March 15, 1977, at Boston University.

41 Jonathan Beckwith and Richard Lewontin on April 11, 1977, at the Harvard Science Center.

42 The Sociobiology Study Group of SftP/Boston University Women’s Center, “Scientific Theories of Innate Differences,” flyer, spring 1977, and: “Are Sex Roles Biologically Determined?,” flyer, spring 1977, Box 888, Folder 1, SJG Papers.

43 Questions for the workshop, Box 888, Folder 1, SJG Papers.

44 “(Male) dominance behavior is natural, genetic, universal among humans and primates. A class-stratified society is biologically programmed. Family structure and behavior are biologically determined. Violence is inevitable because of our biological nature” and the methodological point “We can discover the fundamental aspects of human behavior by the study of behavior of other animals.” Questions for the workshop, Box 888, Folder 1, SJG Papers.

45 “Archaic speculation about human nature is reliable. The things we commonly do, sports, competition in business, our sex role behavior, are natural and healthy. Animals can tell us a lot. We may be over-civilized—it’s time to return to a simpler life based on our ‘true’ nature (cf. current emphasis on back-to-nature foods, clothes etc. Small is beautiful).” Questions for the workshop, Box 888, Folder 1, SJG Papers.

46 “We should tolerate homosexuality because science can now tell us that it’s natural (I’m not sure whether this was explicitly in the film).” It was not, but Wilson repeatedly issued statements to this effect in the press coverage of sociobiology, for example in Wilson (1975b). The worksheet continues: “We need to recognize problems before we can solve them, e.g. an end to all war can come only when we learn how to control our aggressive nature.” Questions for the workshop, Box 888, Folder 1, SJG Papers.

47 “Scientific results (extrapolated a little, of course) confirm our common sense about human nature. It is good to have the courage to seek ‘truth’ in these ‘new’ areas of scientific endeavor.” Questions for the workshop, Box 888, Folder 1, SJG Papers.

48 To what extent does empirical evidence support the claims of sociobiology? Regardless of the degree of support for the claims, is it even reasonable to look for biological (in the sense of the current state of biological research) explanations for complex cultural traits such as aggression, dominance, sexual behavior, etc.? “Is this film unique in its presentation of the biological determinism position, i.e. are there other films, books, etc. past or present that present the position?” Questions for the workshop, Box 888, Folder 1, SJG Papers.

49 “What are the net implications of the biological determinism position regarding warfare, competition, sex roles, race, power and class? Who benefits most from this position? Who does/supports the research that bolsters the biological determinism position?” Questions for the workshop, Box 888, Folder 1, SJG Papers.

50 The minutes also describe other problems within the group: “Moreover, even though active sexism is not so apparent a passive sexism occurs in which the feminist analysis put forward by women is not being taken seriously by men.” Sociobiology Study Group minutes, March 16, 1977, Box 888, Folder 1, SJG Papers.

51 Jon Beckwith of the Boston Group and the Ann Arbor Group were given as contacts in the July/August 1977 issue of *Science for the People Magazine*.

52 Richard Alexander to E. O. Wilson, October 17, 1977, Box 5, EOW Papers.

53 Janet Whitla to Douglas Leiterman, February 22, 1977, Box 186, EOW Papers.

54 Douglas Leiterman to Janet Whitla, March 30, 1977, Box 186, EOW Papers.

55 The EDC/Education Development Center is still a developer and publisher of classroom materials. The legal route with the EDC was taken because Hobel-Leiterman included copyrighted baboon footage shot by DeVore, who transferred the copyright to EDC. As the copyright holder, they were the responsible party for pursuing any violations but argued very much along the lines publicly taken by DeVore, Trivers, and Wilson, when they wrote that they “found many of the images and filmed sequences in Sociobiology that accompanied the spoken comments by scholars to be irrelevant, misleading, and in poor taste in relation to the subject and its implications for the human future.” The sociobiologists used the copyright issue as an “indication of Hobel Leiterman’s irresponsible and apparently deceitful methods.” Leiterman’s response to the letter by the EDC shows this was a genuine legal threat: Regarding the possible copyright infringement, there are no counter-arguments other than claiming the material was used with permission. This is a weak claim without proper documentation. Unsurprisingly, the EDC did not accept the offer to include a disclaimer or credit line. This legal argument was instrumental in removing the original film from circulation by winter 1977. The recut version is, therefore, easy to recognize by the lack of Anubis baboon footage. Janet Whitla to Douglas Leiterman, February 22, 1977; DeVore et al. to *Harvard Crimson*, March 30, 1977, Box 186, EOW Papers.

56 Irven DeVore et al. to *Harvard Crimson*, March 30, 1977, Box 186, EOW Papers.

57 Irven DeVore et al. to *Harvard Crimson*, March 30, 1977, Box 186, EOW Papers.

58 Richard Alexander to E. O. Wilson, October 13, 1977, Box 5, EOW Papers.

59 Richard Alexander to E. O. Wilson, October 13, 1977, Box 5, EOW Papers.

60 The original letter was reprinted in *Anthropology Newsletter* with an introduction by DeVore who seemingly was also taken to task by his anthropological colleagues writing: “Many members of the Association have written to ask me how they could obtain the film Naturally, others have written to chastise me for taking part in a film of such dubious value.” He ends the reprint “I regret whatever cost, inconvenience and embarrassment my colleagues may have suffered by the rental or purchase of this film, and hope they will join us in discouraging its sale and distribution” (DeVore 1977, p. 2,4).

61 His advice was to reprint the letter in journals aimed at colleagues in biology: “Therefore, I think it would be useful if you could make this statement in a more widely read journal like Science or Bioscience.” Richard Alexander to E. O. Wilson, November 14, 1977, Box 5, EOW Papers.

62 Richard Alexander to E. O. Wilson, November 14, 1977, Box 5, EOW Papers.

63 For example, the February 1979 issue of the *Newsletter Animal Behavior Society* states: “Film List: A member of the Film Committee has indicated that a prominent sociobiologist has disavowed the film Naturally (1974) (listed on page 1 of the ABS Film List) as a gross misrepresentation of the field.” A similar denouncement can be found in the spring issue of the Newsletter for the Society of Anthropology of Visual Communication (DeVore 1978, pp. 11–12).

64 A first draft of the review was submitted to the group about a year before its publication. “Tedd was justifiably frustrated by the lack of response to his film critique. He has worked hard on this piece and needs feedback, soon.” Sociobiology Study Group minutes, March 16, 1977, Box 888, Folder 1, SJG Papers.

65 “Whether or not this letter and recalling request would have occurred in the absence of the film’s use by SftP is a matter of speculation” (Judd 1978, p. 16). Given the fact that the first showing was in mid-December and the sociobiologists had organized a legal response by the end of February, it is very likely that one showing would have sufficed to cause the letter and the recalling request.

66 Notable instances were as part of the workshops on sociobiology accompanying the AAAS meeting on February 12–17, 1978, where the infamous water-pouring incident occurred and “several hundred people attended and a good discussion followed” (Beckwith and Lange 1978, p. 39). Stephen Chorover transcribed the film’s soundtrack and transcripts were handed out at the AAAS and other showings (Segerstråle 2000, p. 21, 80). Another was at the National Science Teacher Conference, April 7–9, 1978 in Washington, DC. As always, the showing was an audience hit: “Following the movie, lively dialogue ensued for about an hour” wrote Dan Atkins, Jack Dougherty, Walda Katz Fishman, and Frank Rosenthal in their report of the activities at the conference in the July/August Issue for *Science of the People Magazine* (Atkins et al. 1978, p. 34). SftP’s concern over the influence of sociobiological thinking in education was an especially prominent theme in the controversy as Irven DeVore was connected to the EDC and the development of *Man: A Course of Study*, a controversial course on human nature and behavior (see Milam 2013).

67 When discussing the meanings of the phrase “doing what comes naturally” Evelyn Fox Keller wrote “Recall, for example, the sociobiology film of the same title that *was distributed so widely in the 1970s*, also entitled, Doing what comes naturally” (Keller 2008, p. 124; emphasis added).

68 Letter from Douglas Leiterman to Janet Whitla, March 30, 1977, Box 186, EOW Papers.

69 Letter from John Behnke to Peter Neely, April 5, 1978, Box 186, EOW Papers.

70 “The Sociobiology Debate: A Retrospective,” flyer, 1984, Box 186, EOW Papers.

71 John Tooby (Professor, Department of Anthropology, University of California, Santa Barbara and Co-Director, Center for Evolutionary Psychology), interview by author, Santa Barbara, CA, October 28, 2018.

72 The full tweet reads: “Brings back the memory of Naturally, a 1976 doc released under the auspice of the Sociobiology Study Group (feat. Lewontin), in which heavily edited bits of interviews with Trivers, DeVore, and Wilson were interspersed with shots of napalm and baboon fights,” Twitter, December 12, 2018, tweet no longer online, anonymized screenshot available from the author, emphasis added. The author was a researcher in cognitive science and cultural evolution at the time of posting and tweeted this in reaction to the film release and distribution of *A Dangerous Idea*, directed by Stephanie Welch, Paragon Media Productions, 2016, in which Richard Lewontin, amongst others, warns against the dangers of genetic determinism.

73 For a brief overview of the complicated field encompassing several disciplines such as evolutionary psychology, human behavioral ecology, human ethology, cultural evolution, and gene-culture coevolution, see Mysterud 2004. Current overview by Laland and Brown 2011 and Driscoll 2022.

74 A recent special issue on the influence “Radical Science” (Taylor and Patzke 2021) had on the development of STS as a field illustrates that it is intimately linked to, or founded by, scholars who have first-hand recollections of academic activism, such as resistance to sociobiology.
